# Human papillomavirus type 7 identified in digitated warts on facial seborrhoeic eczema of a non‐butcher immunocompetent individual

**DOI:** 10.1002/ski2.184

**Published:** 2022-11-05

**Authors:** Yuko Izumi, Masaaki Kawase, Kiyofumi Egawa

**Affiliations:** ^1^ Department of Dermatology Jikei University School of Medicine Minato‐ku Tokyo Japan; ^2^ Department of Dermatology Amakusa Dermatology and Internal Medicine Clinic Kami‐Amakusa Kumamoto Japan

## Abstract

Human papillomavirus type 7 (HPV7) is frequently found in butchers' warts and has been demonstrated in several warts of immunocompromised hosts. HPV7 is rarely identified in non‐butchers' warts, especially in individuals with normal immune status. We describe the first case of multiple HPV7‐induced digitated warts which were developed on the face of a 68‐year‐old Japanese man, whose immune status was normal and who had no history of meat handling. Interestingly, the warts were developed exclusively on the skin affected with seborrhoeic eczema in the face, suggesting that some biologically active factors associated with seborrhoeic eczema and anatomical factors of sun‐exposed facial skin might contribute to the development of HPV7‐induced warts.

## Graphical abstract

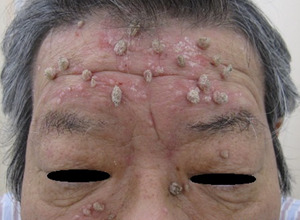

Human papillomavirus type 7 (HPV7) is frequently found in butchers' warts and has been demonstrated in several warts of immunocompromised hosts. We reported the first case of multiple HPV7–induced digitated warts on the seborrhoeic eczema of the face, a sun–exposed area, of a non–butcher immunocompetent individual. In present case, UV exposure might contribute to the activation of the promotor of HPV7 in the face, a sun–exposed area.

## 1 INTRODUCTION

Human papillomavirus type 7 (HPV7) was identified in 1981 in common warts of butchers, and its genome was cloned in 1986 from a wart on the hand of a butcher.^1^, ^2^, ^3^, ^4^

In contrast, HPV7 is rarely in non‐butchers' warts, even though HPV7 has been identified in those of several immuno‐suppressed patients,^5^ or individuals with uncharacterized^6^ and normal immune status,^4^ causing questions in the reservoirs of HPV7 and the route of transmission.^4^, ^6^, ^7^

HPV7 has also been identified in oral warts,^5^ filiform warts of the face,^6^ condyloma of the groin,^8^ or cauliflower‐shaped lesions of the toe‐web.^9^

We describe a patient with HPV7‐induced digitated warts^10^ of the face, whose immune status was normal and who had no history of meat handling. Interestingly, warts developed exclusively on the skin areas affected with seborrhoeic eczema in the face.

## 2 CASE REPORT

A 68‐year‐old man presented to our hospital with a 3‐year history of multiple verrucous papules on the face in November 2010. The papules were developed initially on the forehead and gradually spread to both nasolabial folds of the face.

He was employed as an engineer in telephone equipment and had never worked as a meat handler. He had no different sexual partners including some homosexual contacts in the past. Result of scrutiny, a pathological condition leading to immune‐compromised was not found. He had no history of malignancy.

**FIGURE 1:**
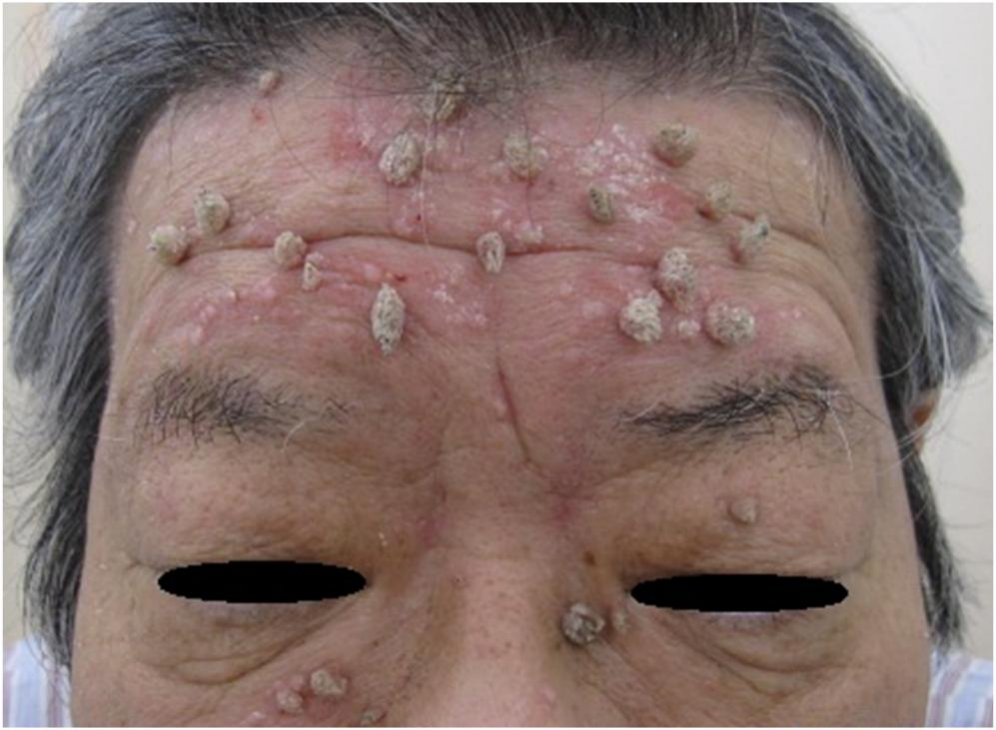
Clinical findings of the lesions. Multiple peculiar verrucous lesions with extreme exophytic growth developing on the facial skin affected with seborrhoeic eczema.

Clinically, peculiar verrucous papules of 5–7 mm diameters showed exophytic growth in the face, with spiky and hyperkeratotic surfaces to be diagnosed with the digitated wart. Interestingly, the verrucous papules developed exclusively on the scaly and erythematous skin lesions diagnosed with seborrhoeic eczema^11^ (Figure 1). Common wart‐like papules were also seen on the left hand.

After informed consent was obtained, two biopsies were taken from a verrucous papulae of the face and scaly erythema surrounding the papulae under local anaesthesia with the diagnosis of the digitated wart and seborrhoeic eczema.

**FIGURE 2:**
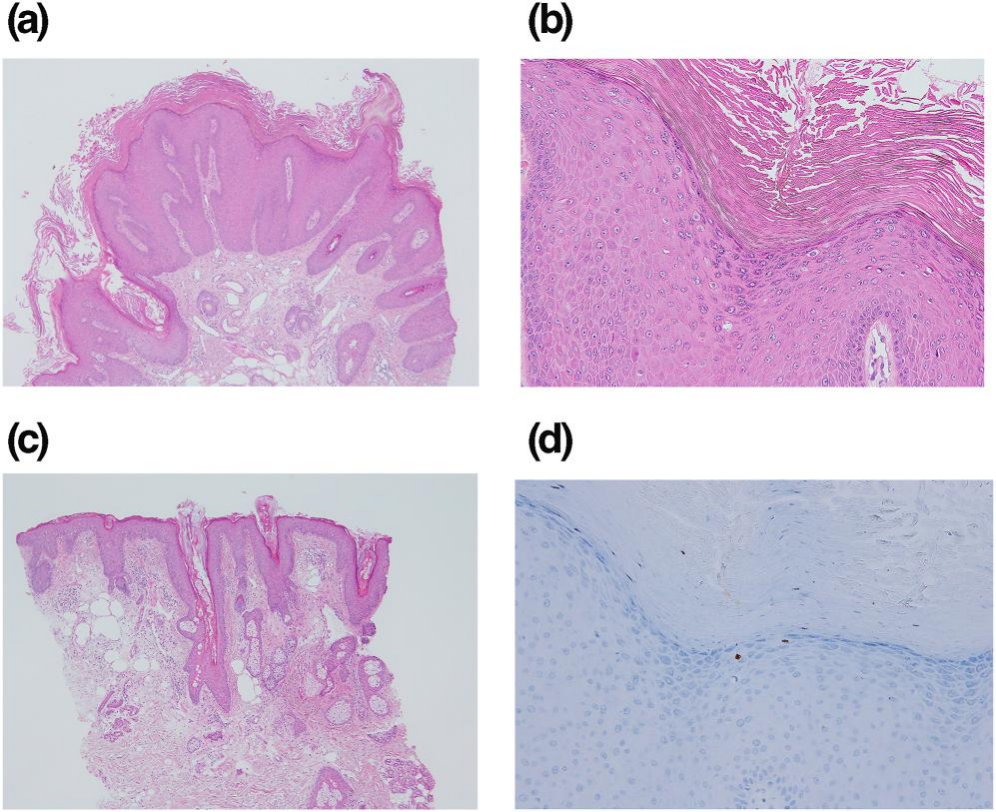
Histological and immunohistochemical findings of the lesions. (a) Histological findings of the lesions of the wart (haematoxylin and eosin (HE), x100). The epidermis shows acanthosis with papillomatosis, hyperkeratosis and focal parakeratosis. (b) Histological findings of the lesions of the wart (HE, x200). Vacuolated cells with centred nuclei, in which no keratohyalin granule, are seen in the upper epidermis. (c) Histological findings of the skin surrounding warts (HE, x100). The epidermis in the follicular ostium shows irregular acanthosis with relatively thin parakeratotic horny layer including neutrophils. Spongiosis is found, but Munro's micro abscess is not found. No papillomatosis is found in the epidermis. The upper dermis shows perivascular lymphocytic infiltration. (d) Immunohistochemistry for HPV antigens of the lesions of the wart (x200). The nuclei of vacuolated cells scattered in the upper epidermal cell layers are positive for K1H8 anti‐papillomavirus monoclonal antibody.

Histological examination with haematoxylin and eosin (HE) stain showed the verrucous papulae to be a common wart^10^ (Figure 2a); the epidermis showed acanthosis with papillomatosis, hyperkeratosis, and focal parakeratosis. A few clear cells were seen in the granular layer, which has centred nuclei and no keratohyalin granule (Figure 2b). Skin lesions surrounding warts showed findings compatible with those of seborrhoeic eczema^11^ (Figure 2c). The epidermis in the follicular ostium showed irregular acanthosis with a relatively thin parakeratotic horny layer including neutrophils. Spongiosis was found, but Munro's micro abscess was not found. No papillomatosis was found in the epidermis. The upper dermis showed perivascular lymphocytic infiltration.

Immunohistochemically, using avidin‐biotin‐peroxidase complex (ABC) method^12^ with K1H8 (DAKO) anti‐papillomavirus monoclonal antibody, positive signals were seen in the nuclei of the vacuolated cells in the epidermis of the papulae (Figure 2d), while no particular reactivity was seen in the surrounding skin of seborrhoeic eczema (data not shown).

**FIGURE 3:**
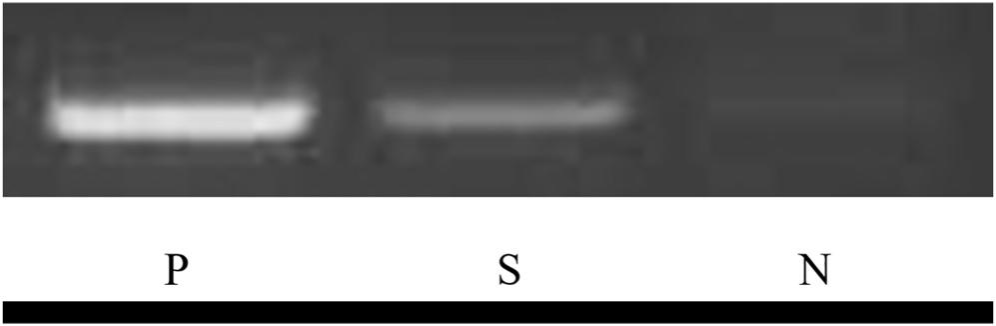
HPV7‐related DNA detection using polymerase chain reaction MK7F/MK7R primer. N, Negative control; P, Positive control (human papillomavirus type 7 (HPV7) genome); S, Sample

HPV polymerase chain reaction (PCR) in the extracted DNA from the wart (fresh material) was performed with the specific primers allowing the detection of the five main skin‐related HPV types (HPV‐1, ‐2, ‐3, ‐4, ‐7) (Table 1). The only PCR product using MK7F/MK7R primer was positive (Figure 3). The PCR product was analyzed by direct sequencing, which revealed that the sequence corresponded to the L1 gene of HPV type7 (Genebank: X74463).

**TABLE 1:** Alignment of specific primers for amplification of a conserved region within the L1 open reading frame of HPV

Detectable HPV types	Primer name^a^	Sequence (5′ to 3′)	Position in HPV type^b^	Lenth of PCR product^c^
1, 63	MK1F	TGCAAATATCCTGATTATATCAGAATG	nt 6109‐6135 in HPV1	251bp
	MK1R	CTATTAAACAATTGGACATCAGA	nt 6338‐6360 in HPV1	
2, 27, 57	MK2F	ACTCCTAGTGGCTCTATGGTGTCCTCTGAA	nt 6654‐6683 in HPV2	313bp
	MK2R	AACAACTGGGGATCCATATTATGTAT	nt 6942‐6967 in HPV2	
3, 10, 28, 94	MK3F	AATAAGCCATATTGGCTGCGGCG	nt 6662‐6684 in HPV3	341bp
	MK3R	GTGTCCTCCAAGCTAGTGGA	nt 6984‐7003 in HPV3	
4, 60, 65	MK4F	TTAATCGACCGTATTGGTTAAACAGAG	nt 6290‐6137 in HPV4	269bp
	MK4R	CATTACATTTAAATGAGC	nt 6542‐6559 in HPV4	
7, 40	MK7F	GGAATGGCTGCAGAACCGTATGG	nt 6497‐6519 in HPV7	240bp
	MK7R	CCTTTTGTATCCACAA	nt 6722‐6737 in HPV7	

Abbreviation: PCR, polymerase chain reaction.

^a^ F, forward; R, reverse.

^b^ nt, nucleotide.

^c^ bp, base pair.

Aside from the multiple warts of the face and the left hand and seborrhoeic eczema of the face, he was otherwise healthy. Routine laboratory parameters yielded normal values.

Under the diagnosis of HPV7‐induced digitated warts, he was treated with liquid nitrogen cryotherapy, resulting in complete resolution 7 months later. For seborrhoeic eczema, we applied ketoconazole cream twice a day. Along with the disappearance of warts, seborrhoeic eczema also disappeared. To date, 10 years after completion of the therapy, the patient is relapse‐free.

## 3 DISCUSSION

While HPV7 is frequently identified in butchers' warts, HPV7 is rarely identified in non‐butchers' warts, especially in individuals with normal immune status.

Keefe et al.^4^ studied 240 abattoir workers, 246 retail and wholesale butchers, 308 engineering fitters, and 292 office workers, revealing that the prevalence of hand warts was 33.3% in the abattoir workers, 34.1% in the butchers, 19.5% in the engineers and 14.7% in the office workers and that the prevalence of HPV7 was 15.6% in the abattoir workers, 15.5% in the butchers, 0% in the engineers and 0.6% in the office workers.

Apart from common warts/common wart‐like lesions^1^, ^2^, ^3^, ^4^, ^13^ of hands of butchers, HPV7 has rarely been identified in oral warts,^5^ filiform warts of the face,^6^ or condyloma of the groin^8^ of immunocompetent as well as immunocompromised individuals of non‐butcher, which has caused questions in the reservoirs of HPV7 and the route of transmission,^4^, ^6^, ^7^ or the clinical morphology HPV7 could induce, other than butchers' warts.^8^ Cauliflower‐shaped lesions of the toe‐web have proposed a new clinical entity associated with HPV7.^9^

The route of infection was also unclear in our patient, but it was quite interesting that the HPV7‐induced warts developed exclusively on the skin affected with seborrhoeic eczema in the face, suggesting that some unknown factors associated with seborrhoeic eczema might contribute to the development of warts.

de Villiers et al.^14^ deduced that HPV7 could exist latently in the skin of a normal population and be reactivated by some unknown factors to develop clinically identifiable lesions. Keefe et al.^4^, ^7^ speculated for HPV7‐induced butchers' warts that meat has factors to enhance the ability of HPV7 to replicate in the keratinized epithelium. Immunosuppression should also be a factor since HPV7‐induced warts have been reported in several cases of HIV‐infected patients.^5^

de Villiers et al. also demonstrated activation of the promoter of some HPV types, including HPV7, under the influence of UV irradiation.^14^ In the present case, UV exposure might also contribute to the activation of the promotor of HPV7 in the face, a sun‐exposed area.

In conclusion, it seems likely that HPV7 infection is widespread but latent among people and some factors, such as that associated with meat in case of butchers or immunodeficiency in case of HIV‐infected patients, activate HPV7 to produce clinically visible lesions. For HPV7‐associated warts in the toe‐web, it is speculated that the long‐standing tinea pedis and the wet environment in the interdigital areas played an important role in HPV 7 infection and proliferation. In the present case, factors associated with seborrhoeic dermatitis and sun exposure might play such a role. It is also possible that the oral mucosa served as a reservoir of HPV 7^5^ for productive infection of seborrhoeic facial skin, although the buccal mucosa of the present case was not analyzed for the presence of HPV 7.

Concerning the clinical features of HPV7‐induced warts, they could also vary depending on the anatomical factors from common warts in hands, oral warts, filiform/digitated warts in the face, genital warts, or cauliflower‐shaped lesions in the toe‐web, as is the case for other HPV types.^8^, ^15^

## CONFLICT OF INTEREST

The authors declared that they have no conflicts of interest to this work.

## AUTHOR CONTRIBUTIONS

**Yuko Izumi**: Conceptualization (Lead); Data curation (Lead); Formal analysis (Equal); Investigation (Equal); Methodology (Equal); Project administration (Equal); Resources (Equal); Validation (Lead); Visualization (Equal); Writing – original draft (Lead). **Masaaki Kawase**: Conceptualization (Supporting); Data curation (Supporting); Formal analysis (Equal); Investigation (Equal); Methodology (Equal); Project administration (Equal); Resources (Equal); Supervision (Lead); Validation (Supporting); Visualization (Equal); Writing – review & editing (Supporting). **Kiyofumi Egawa**: Supervision (Supporting); Writing – review & editing (Lead).

## ETHICS STATEMENT

We confirmed the patient consent for the publication of all case reports and all clinical images, and that appropriate consent has been obtained.

## Data Availability

The data that support the findings of this study are available from the corresponding author upon reasonable request.
